# Macroscopic and microscopic assessments of the glenohumeral and subacromial synovitis in rotator cuff disease

**DOI:** 10.1186/s12891-015-0740-x

**Published:** 2015-09-30

**Authors:** Chris H. Jo, Ji Sun Shin, Ji Eun Kim, Sohee Oh

**Affiliations:** Department of Orthopedic Surgery, SMG-SNU Boramae Medical Center, Seoul National University College of Medicine, Seoul, Korea; Department of Pathology, SMG-SNU Boramae Medical Center, Seoul National University College of Medicine, Seoul, Korea; Department of Biostatistics, SMG-SNU Boramae Medical Center, Seoul, Korea

## Abstract

**Background:**

Whereas synovitis is one of most common findings during arthroscopic surgery in patients with rotator cuff diseases, no study has investigated its characteristics. We propose a macroscopic assessment system for investigating the characteristics of synovitis.

**Methods:**

Fifty-four patients with a full-thickness rotator cuff tear undergoing arthroscopic rotator cuff repair with an average age of 62.5 ± 7.0 years were included. For the macroscopic assessment, 3 parameters, villous hypertrophy, hyperemia, and density, were measured and translated into grades in 3 regions-of-interest (ROI) in the glenohumeral joint and 4 ROIs in the subacromial space. For the microscopic assessments, 4 commonly used microscopic assessment systems were used. The reliability and association between the macroscopic and microscopic assessments were investigated.

**Results:**

The inter- and intra-observer reliability of all of the macroscopic and microscopic assessments were excellent. The severity of synovitis was significantly greater in the glenohumeral joint than that in the subacromial space, 1.54 ± 0.61 versus 0.94 ± 0.56 (*p* < 0.001). Synovitis varied with respect to location, and was generally more severe near the tear with the macroscopic assessment system. Meanwhile, none of the microscopic assessment systems demonstrated differences between different ROIs in both the glenohumeral joint and the subacromial space.

**Conclusions:**

The macroscopic assessment system for synovitis in rotator cuff disease in this study showed excellent reliability. It critically described characteristics of synovitis that microscopic assessment systems could not. Therefore, this system could be a useful tool for investigating synovitis in rotator cuff disease.

## Background

Shoulder pain is reported to be the third most common musculoskeletal disorder (16 %), after back (23 %) and knee pain (19 %) [1–3], and it poses a substantial socioeconomic burden of up to $7 billion in the United States [4]. Approximately 40–50 % of patients will still have persistent pain even after 1 year of conservative treatment [5, 6]. Rotator cuff disease is the most common cause of shoulder pain [7–9]. The prevalence of symptomatic rotator cuff disease increases with age, occurring in approximately 2.8 % of those older than 30 years and in 15 % of those older than 70 years [7, 10]. In the United States, rotator cuff diseases lead to more than 4.5 million yearly physician visits, and over 300,000 rotator cuff repairs per year are performed annually costing more than $3 billion [11, 12].

Rotator cuff disease, or syndrome, represents a spectrum of rotator cuff pathologies from subacromial bursitis or tendinopathy, partial- and full-thickness tear, and rotator cuff arthropathy [13]. Subacromial bursitis and tendinopathy are also called as impingement syndrome [14]. Meanwhile, it is well known that rotator cuff disease involves not only the rotator cuff tendon but also rotator cuff muscles proximally [15], tissues of the glenohumeral joint and subacromial space, including synovium, ligaments, labrum and bursa in the middle [16, 17]; and the proximal humerus distally [18]. Therefore, the authors suggest that rotator cuff disease needs be regarded and treated as a “pan-joint disease” of the shoulder similar to osteoarthritis [19].

Among the pathologic changes observed during rotator cuff surgery, synovitis in the glenohumeral joint and subacromial space is one of most frequently observed findings. As evidences in rheumatoid arthritis and osteoarthritis have shown that synovitis is associated with exacerbated symptoms such as pain and degree of joint dysfunction [20, 21], and that it may promote cartilage degeneration, it is not difficult to assume that synovitis may also have certain roles in rotator cuff disease. The first step in the management of synovitis in rotator cuff disease should be the establishment of a reliable method for describing characteristics and monitoring the severity of synovitis. Except for some laboratory studies reported on synovial inflammation in the subacromial bursa as a pain source [22–29], and as a factor for rotator cuff degeneration [30], few studies have reported the characteristics of synovitis in rotator cuff disease [29, 31, 32]. Microscopic assessment is usually considered the gold standard for analysis of synovitis in osteoarthritis [33]. However, microscopic assessments might not be feasible in some clinics, would be hard to cover different regions in the joint and subacromial space, could not provide direct information at the time of surgery, and has been reported to fail to detect a relationship between that and pain or disability in osteoarthritis [33]. In this sense, whereas a macroscopic assessment system of the characteristics of synovitis in rotator cuff disease would be helpful, no study has suggested any tool for macroscopic evaluation of the characteristics of synovitis in rotator cuff disease.

Therefore, the purposes of the study were to propose and validate a macroscopic assessment system for synovitis in the glenohumeral joint and subacromial space in rotator cuff disease and to investigate the characteristics of synovitis according to this system.

## Methods

### Study design and patients

This prospective cohort study was approved by our institutional review board (SMG-SNU Boramae Medical Center Institutional Review Board), and all patients provided informed consent. Eligible patients were those with a full-thickness rotator cuff tear and available tissue samples from both glenohumeral and subacromial synovium harvested at the time of surgery. We excluded patients if they had impingement disease, a partial-thickness tear, rotator cuff arthropathy, infection, isolated subscapularis tear, calcific tendinitis, retear, or no available tissue samples.

We proposed a macroscopic assessment system for synovitis of the glenohumeral joint and subacromial space in patients with a full-thickness rotator cuff tear. For validation of the macroscopic assessment system, inter- and intra-observer reliability tests were conducted and associations with 4 commonly used microscopic assessment systems were analyzed.

### ROI in the glenohumeral joint and subacromial space

**Fig. 1:**
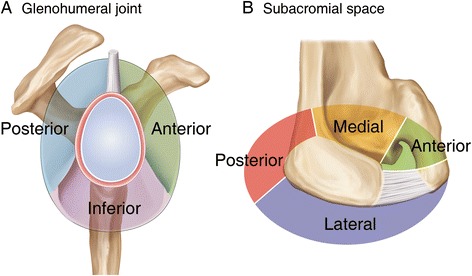
Regions-of-interest (ROIs) in the glenohumeral joint and subacromial space. **a** Synovium in the glenohumeral joint was divided into 3 ROIs; the anterior, posterior, and inferior synovium. The anterior glenohumeral joint synovium was outlined by the long head of biceps superiorly and by the anterior band of the inferior glenohumeral ligament inferiorly. The inferior glenohumeral joint synovium was defined by the anterior and posterior bands of the inferior glenohumeral ligament. The posterior glenohumeral joint synovium was located by the posterior bands of the inferior glenohumeral ligament inferiorly and by the long head of biceps superiorly. **b** Synovium in the subacromial space was divided into 4 ROIs; the anterior, posterior, medial, and lateral synovium. The anterior subacromial synovium was outlined by the posterior margin of the coracoacromial ligament laterally and by the base of the coracoid medially. The medial subacromial synovium was defined by the base of the coracoid anteriorly and by the base of the scapular spine posteriorly. The posterior subacromial synovium was located by the base of the scapular spine medially and by the posterolateral angle of the acromion laterally. The lateral subacromial synovium was determined by the posterolateral angle of the acromion posteriorly and the anterior margin of the coracoacromial ligament anteriorly

The macroscopic and microscopic assessments were performed in the 3 ROIs of the glenohumeral joint (anterior, inferior, and posterior), and in the 4 ROIs of the subacromial space (anterior, posterior, medial, lateral) for each patient (Fig. 1).

### Macroscopic assessment of synovitis with arthroscopy

All procedures were performed in the lateral decubitus position under general anesthesia as previously described [34]. After systematic exploration of the glenohumeral joint, the macroscopic assessment of synovitis in the anterior, posterior and inferior ROIs was performed. Synovial tissue was harvested from each ROI using a basket forceps. Then, the arthroscope was removed and redirected to the subacromial space. A lateral working portal and a posterolateral viewing portal were also established. Exploration of the subacromial space and the assessment of synovitis were performed in the anterior, posterior medial and lateral ROIs, followed by harvesting of the synovial tissues from each ROI.

Macroscopic assessment of synovitis were performed with three complementary parameters with respect to synovial villi in each ROI; hypertrophy, hyperemia, and density. Only villi groups with 5 or more villi were considered, and any isolated group with fewer than 5 villi was excluded. If arthroscopically different-looking groups of villi were simultaneously observed in the same ROI, each was graded and the worse grade was selected. Hypertrophy was scored based on the size of the synovial villi; 0, < 2 mm; 1, 2 ~ 5 mm; 2, > 5 mm. Hyperemia represents the vascularity of synovial villi and was evaluated based on the redness of the villi; 0, pale and transparent; 1, slightly reddish; 2, definitely red. If not apparent, hyperemia was determined with the harvested synovial tissue. Density was assessed by the coverage of synovial villi in each ROI; 0, < 1/3; 1, 1/3 ≤. After adding the scores for each parameter, the macroscopic grade of synovitis was defined as follows; grade 0, 0; grade 1, 1–2; grade 2, 3; grade 3, 4. Macroscopic assessment of synovitis was conducted by two fellowship-trained orthopedic surgeons independently.

### Microscopic assessment of synovitis

**Fig. 2:**
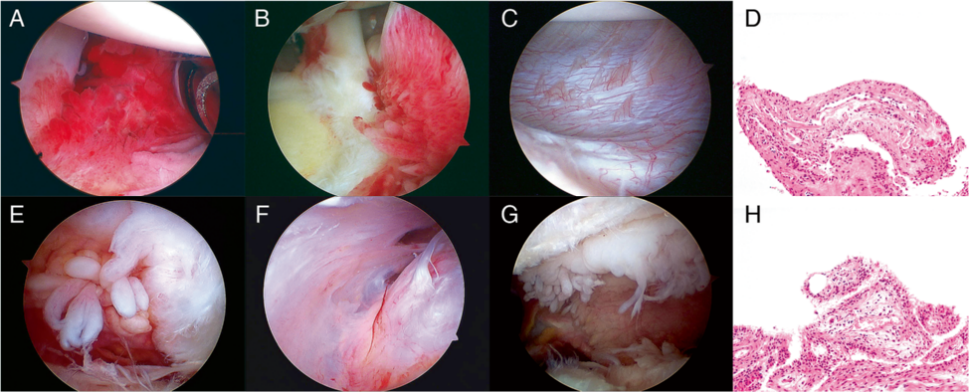
Macroscopic and microscopic findings of synovitis in the glenohumeral joint (*upper row*) and subacromial space (*lower row*) with arthroscopy. **a** The anterior synovium in the glenohumeral joint. The macroscopic assessment was villous hypertrophy, 1; hyperemia, 1; density, 1; grade, 2. **b** The posterior synovium in the glenohumeral joint. Villous hypertrophy, 1; hyperemia, 1; density, 1; grade 2. **c** The inferior synovium in the glenohumeral joint. Villous hypertrophy, 0; hyperemia, 0; density, 0; grade, 0. **d** The microscopic findings of the anterior synovium in the glenohumeral joint. The macroscopic assessment was villous hypertrophy, 1; hyperemia, 1; density, 1; grade 2. The microscopic assessments were the Østergaard grade, 1.7; the Loeuille grade, 1.7; the modified Krenn grade, 2.0; the Scanzello grade, 2.0. **e** The anterior synovium in the subacromial space. The macroscopic assessment was villous hypertrophy, 2; hyperemia, 0; density, 1; grade, 2. **f** The posterior synovium in the subacromial space. Villous hypertrophy, 0; hyperemia, 0; density, 0; grade 0. **g** The lateral synovium in the subacromial space. Villous hypertrophy, 2; hyperemia, 0; density, 0; grade, 1. **h** The microscopic finding of the lateral synovium in the subacromial space. The macroscopic assessment was villous hypertrophy, 2; hyperemia, 0; density, 1; grade 2. The microscopic assessments were the Østergaard grade, 1.3; the Loeuille grade, 1.2; the modified Krenn grade, 2.0; the Scanzello grade, 2.0

Biopsy specimens were immediately fixed in neutral buffered 10 % formalin. Subsequently, the specimens were embedded in paraffin, sectioned, and stained with hematoxylin and eosin (H&E). Microscopic assessment was performed according to 4 commonly used microscopic assessment systems for synovitis; the Østergaard, Loeuille, modified Krenn, and Scanzello systems (Fig. 2) [21, 35–37]. For the Østergaard grade [21], the following parameters were used: 1) subsynovial infiltration of polymorphonuclear leucocytes; 2) subsynovial infiltration of mononuclear leucocytes; 3) surface fibrin deposition; 4) multiplication of the synovial lining; 5) villous hypertrophy of the synovial surface; 6) proliferation of blood vessels; 7) perivascular edema; 8) formation of granulation tissue; 9) fibrosis. For the Loeuille grade [37], six parameters were examined: 1) number of synovial lining cells; 2) subsynovial infiltration by lymphocytes and plasma cells; 3) surface fibrin deposition; 4) congestion related to blood vessel vasodilatation and, to a minor degree, blood vessel proliferation; 5) fibrosis, and 6) perivascular edema. For the modified Krenn grade [36], three parameters were included: 1) synovial lining layer, 2) degree of inflammatory infiltration, and 3) activation of resident cells and synovial stroma including fibroblasts, endothelial cells, histiocytes, macrophages, and multinucleated giant cells. For the Scanzello grade [35], the synovial inflammation was graded based on perivascular mononuclear cell infiltration in synovium; 0 non, 1, 0–1 perivascular aggregates per low-power field, 2, > 1 perivascular aggregate per low-power field with or without focal interstitial infiltration, 3 marked aggregates both perivascular and interstitial.

An experienced pathologist and an orthopedic surgeon, both of whom were blinded to the harvest site, evaluated the stained sections. Parameters of each assessment system were scored 0 none, 1 mild, 2 moderate, or 3 severe. The average grade of the parameters was calculated and used as the respective grade.

### Statistical analyses

To determine the study sample size, an *a priori* power analysis was performed to provide a statistical power of 90 % at an alpha level of 0.05. Our pilot study with 30 patients showed that the Pearson correlation coefficient between the macroscopic and microscopic assessments was 0.465 with a statistical power of 75.8 % (*p* = 0.010). Using a sample size calculation for correlation, a sample size of 54 patients per group was determined assuming data loss of 10 %. Power analysis and sample size software (NCSS 2005; NCSS, Kaysvill, UT, USA) was used for this calculation. Inter- and intra-observer reliability were assessed with the intraclass correlation coefficients (ICCs) for the macroscopic and microscopic measures. Second examination for the intra-observer reliability was done 1 month after the first examination. The paired *t*-test was used to compare the macroscopic and microscopic measures. One-way analysis of variance (ANOVA) was used to compare the macroscopic and microscopic measures between ROIs in the glenohumeral joint and the subacromial space. Associations between the parameters of the macroscopic assessments and the microscopic assessments were examined by calculating the Spearman correlation coefficient, and associations between the macroscopic grade and the microscopic assessments were analysed by the Pearson correlation. All analyses were performed using SPSS version 13.0 (SPSS Inc., Chicago,

## Results

### Patient demographics

From January 2012 to April 2013, 233 patients with rotator cuff disease underwent arthroscopic surgery. Among them, 179 were excluded; 110 without glenohumeral or subacromial synovium, 25 with partial-thickness rotator cuff tear, 12 with arthropathy, 8 with infection, 8 with impingement disease, 7 with isolated subscapularis tear, 6 with calcific tendinitis, and 3 with retear. Therefore, 54 patients were included in the study.

Generally, the patients included in the study appeared to represent common features of patients undergoing rotator cuff repair. The mean age was 62.5 ± 7.0 years with average symptom duration of 15.0 months (Table 1). There were 16 males (29.6 %) and 38 females (70.4 %). The medium tear (57.4 %) and stage II retraction (38.9 %) were most frequent, respectively. The majority of patients had grade B (53.7 %) for the visual tendon grade, and minimal osteoarthritic change of Kellgren-Lawrence grade 0 (18.5 %) or 1 (68.5 %). The mean global fatty degeneration index (GFDI) was 1.7, suggesting mild to moderate chronicity of tear. Common associated lesions included superior labral anterior and posterior (SLAP) lesions (37.0 %), subscapularis tear (77.8 %), and biceps tear (42.6 %).

**Table 1:** Baseline characteristics of patients

Characteristics^a^	Value
Age^b^ (yr)	62.5 ± 7.0
Sex, M:F	16:38
Dominance, Y:N	47:7
Duration^b^ (mo)	15.0 ± 21.0
Cofield type, small:medium:large:massive	4:31:11:8
Boileau stage, I:II:III:IV	18;21:10:5
Tendon grade, A:B:C^c^	21:29:4
Kellgren-Lawrence grade, 0:1:2:3:4^d^	10:37:7:0:0
GFDI^b^	1.7 ± 0.7
Associated lesions	
SLAP lesion	34 (63.0 %)
Subscapularis tear	14 (25.9 %)
Biceps tear	6 (11.1 %)

^a^ *GFDI* global fatty degeneration index, *SLAP* superior labrum anterior and posterior

^b^The values are given the mean and the standard deviation

^c^Tendon grade assesses rotator cuff quality using 3 gross tendon criteria: (1) fraying over half of the tendon thickness, (2) delamination of the supraspinatus tendon, and (3) thinning of less than half of the normal thickness. A, none of these criteria were met; B, fraying or delamination was identified; C, both fraying and delamination or thinning regardless of the other criteria

^d^Kellgren-Lawrence grade evaluates the radiographic severity of osteoarthritis of the knee: Grade 0, normal; grade 1, doubtful narrowing of the joint space and possible osteophyte lipping (irregular bone formation); grade 2, definite osteophytes and possible narrowing of the joint space; grade 3, multiple moderate-size osteophytes, definite narrowing of the joint space, some sclerosis, and possible deformity of bone contour; grade 4, large osteophytes, marked narrowing of the joint space, severe sclerosis, and definite deformity of bone contour

### Inter- and intra-observer reliability of the macroscopic and microscopic assessments

The inter- and intra-observer reliability of all of the macroscopic and microscopic assessments were excellent (Table 2). All of the measured ICCs were above 0.8. Among the 3 parameters of the macroscopic assessments, the highest inter- and intra-observer reliability were found for hyperemia (0.930) and density (0.941), respectively. Among the 4 microscopic grading systems, the Scanzello grading system showed the highest inter-observer reliability (0.942), and the Østergaard had the highest intra-observer reliability (0.931).

**Table 2:** Inter- and intra-observer reliability of the macroscopic and microscopic assessments

	Inter-observer			Intra-observer		
	ICC	95 % CI^a^	*p* value	ICC	95 % CI	*p* value
Macroscopic parameters						
Hypertrophy	0.936	(0.915, 0.951)	< .001	0.960	(0.948, 0.969)	< .001
Hyperemia	0.935	(0.916, 0.950)	< .001	0.885	(0.851, 0.911)	< .001
Density	0.955	(0.941, 0.966)	< .001	0.962	(0.950, 0.971)	< .001
Macroscopic grade	0.949	(0.933, 0.961)	< .001	0.959	(0.947, 0.969)	< .001
Microscopic assessment						
Østergaard	0.924	(0.900, 0.942)	< .001	0.947	(0.931, 0.959	< .001
Loeuille	0.910	(0.882, 0.932)	< .001	0.931	(0.911, 0.947)	< .001
Modified Krenn	0.948	(0.932, 0.961)	< .001	0.969	(0.960, 0.977)	< .001
Scanzello	0.884	(0.848, 0.912)	< .001	0.973	(0.964, 0.979)	< .001

^a^ *ICC* intraclass correlation, *CI* confidence interval

### Macroscopic assessment of synovitis in the glenohumeral joint and subacromial space

The average scores of the macroscopic parameters, villous hypertrophy, hyperemia and density, were significantly higher in the glenohumeral joint than those in the subacromial space (Table 3). The average macroscopic grade of synovitis was also significantly greater in the glenohumeral joint than that in the subacromial space; 1.54 ± 0.61 versus 0.94 ± 0.56 (*p* < 0.001).

**Table 3:** Macroscopic and microscopic assessments of synovitis in the glenohumeral joint and subacromial space

	Glenohumeral joint^a^					
	Anterior	Posterior	Inferior	p value	Average^b^	*p* value*
Macroscopic parameter						
Hypertrophy	1.22 ± 0.60	1.30 ± 0.66	1.06 ± 0.68	.148	1.19 ± 0.50	< .001
Hyperemia	0.50 ± 0.50	0.57 ± 0.50	0.22 ± 0.42	< .001	0.43 ± 0.34	< .001
Density	0.67 ± 0.48	0.83 ± 0.38	0.59 ± 0.50	.020	0.70 ± 0.33	< .001
Macroscopic grade	1.56 ± 0.79	1.81 ± 0.78	1.26 ± 0.65	.001	1.54 ± 0.61	< .001
Microscopic assessment						
Østergaard	1.18 ± 0.52	1.20 ± 0.49	1.16 ± 0.35	.878	1.18 ± 0.46	.002
Loeuille	1.44 ± 0.58	1.49 ± 0.54	1.46 ± 0.41	.890	1.46 ± 0.51	.002
m. Krenn	1.43 ± 1.06	1.52 ± 0.84	1.25 ± 0.72	.269	1.40 ± 0.88	.019
Scanzello	1.17 ± 1.09	1.50 ± 1.04	1.31 ± 1.04	.265	1.33 ± 1.06	.390
	Subacromial space^a^					
	Anterior	Posterior	Medial	Lateral	*p* value	Average^c^
Macroscopic parameter						
Hypertrophy	0.78 ± 0.77	0.41 ± 0.63	0.80 ± 0.74	1.02 ± 0.74	< .001	0.75 ± 0.47
Hyperemia	0.19 ± 0.39	0.09 ± 0.29	0.19 ± 0.39	0.20 ± 0.41	.406	0.17 ± 0.27
Density	0.46 ± 0.50	0.20 ± 0.41	0.59 ± 0.50	0.56 ± 0.50	< .001	0.45 ± 0.32
Macroscopic grade	1.00 ± 0.77	0.59 ± 0.84	0.94 ± 0.66	1.24 ± 0.78	< .001	0.94 ± 0.56
Microscopic assessment						
Østergaard	1.03 ± 0.29	1.03 ± 0.27	1.07 ± 0.37	0.94 ± 0.35	.101	1.04 ± 0.31
Loeuille	1.27 ± 0.37	1.31 ± 0.30	1.33 ± 0.45	1.20 ± 0.42	.166	1.30 ± 0.38
m. Krenn	1.17 ± 0.60	1.07 ± 0.64	1.33 ± 0.79	1.21 ± 0.70	.269	1.19 ± 0.68
Scanzello	1.26 ± 1.05	1.07 ± 0.95	1.37 ± 0.81	1.15 ± 0.79	.136	1.23 ± 0.94

*Comparison between the averages of the macroscopic and microscopic measures of the glenohumeral joint and the subacromial space using the paired *t*-test

^a^The values are given the mean and the standard deviation

^b^Average of the macroscopic measures in the glenohumeral joint

^c^Average of the macroscopic measures in the subacromial space

The severity of synovitis was different with respect to location, and synovitis was greatest near the rotator cuff tear and least away from the tear in both the glenohumeral joint and the subacromial space. The grades of the posterior and anterior synovium, 1.81 ± 0.78 and 1.56 ± 0.79, respectively, were significantly higher than that of the inferior synovium, 1.26 ± 0.65, in the glenohumeral joint. The grade of the lateral synovium, 1.24 ± 0.78, was significantly higher than those of the medial and posterior synovium, which were 0.94 ± 0.66 and 0.59 ± 0.84, respectively. The posterior synovium showed exceptionally lower severity in every parameter and grade of synovitis assessment.

The differences in the macroscopic assessment mainly resulted from the differences of villous hyperemia or density rather than hypertrophy in both the glenohumeral joint and the subacromial space (Table 3). Hypertrophy in the glenohumeral joint was not different with respect to ROI (*p* = 0.148). Whereas hypertrophy in the subacromial space was different among 4 ROIs (*p* < 0.001), those of 3 ROIS except for the posterior synovium were not different, suggesting similar results to those in the glenohumeral joint.

### Microscopic assessments of synovitis in the glenohumeral joint and subacromial space

Synovitis measured with the Østergaard, Loeuille, and modified Krenn grading systems showed that the severity of synovitis in the glenohumeral joint was significantly higher than that in the subacromial space (Table 3). The Scanzello grading system did not show a significant difference (*p* = 0.390).

There was no significant difference in synovitis between the different ROIs in both the glenohumeral joint and the subacromial space measured with any of the 4 microscopic grading systems (Table 3).

### Association between the macroscopic and microscopic assessments

The associations between the macroscopic grades and the microscopic assessments were stronger in the glenohumeral joint than in the subacromial space (Table 4 and 5). The strongest association between the macroscopic and microscopic assessments was found with the Østergaard system in both the glenohumeral joint (*r* = 0.683) and the subacromial space (*r* = 0.515).

**Table 4:** Association between the macroscopic and microscopic assessments of synovitis in the glenohumeral joint and subacromial space

	Glenohumeral joint							
	Anterior		Posterior		Inferior		Average	
	r	p value	r	p value	r	p value	r	p value
Microscopic assessment								
Østergaard	0.502	<.001	0.661	<.001	0.440	.002	0.683	<.001
Loeuille	0.489	<.001	0.650	<.001	0.384	.012	0.647	<.001
m. Krenn	0.541	<.001	0.693	<.001	0.369	.008	0.675	<.001
Scanzello	0.478	<.001	0.605	<.001	0.295	.009	0.564	<.001

**Table 5:** Association between the macroscopic and microscopic assessments of synovitis in the glenohumeral joint and subacromial space

	Subacromial space									
	Anterior		Posterior		Medial		Lateral		Average	
	r	p value	r	p value	r	p value	r	p value	r	p value
Microscopic assessment										
Østergaard	0.165	.233	0.219	.112	0.304	.025	0.215	.118	0.515	<.001
Loeuille	0.044	.753	0.233	.090	0.279	.041	0.178	.197	0.500	<.001
m. Krenn	0.243	.077	0.045	.744	0.182	.188	0.217	.115	0.370	.006
Scanzello	0.370	.006	0.134	.334	0.111	.426	0.342	.011	0.380	.005

The strength of the association between the macroscopic and microscopic assessments varied with respect to ROI and the type of microscopic assessments in both the glenohumeral joint and the subacromial space. In the glenohumeral joint, the strongest association was found in the posterior synovium, followed by the anterior and inferior synovium regardless of the type of microscopic assessment. The modified Krenn system showed the highest association in the anterior (*r* = 0.541) and posterior synovium (*r* = 0.693), and the Østergaard system showed the highest association in the inferior synovium (*r* = 0.440). In the subacromial space, the strongest association varied widely according to the ROI and the type of microscopic assessment. There was no significant association in the posterior synovium with any of the 4 microscopic assessments. The Scanzello system showed the highest association in the anterior (*r* = 0.370) and lateral synovium (*r* = 0.342), and the Østergaard system showed the highest association in the medial synovium (*r* = 0.304).

## Discussion

In this study, we proposed a macroscopic assessment system for the evaluation of synovitis in patients with a full-thickness rotator cuff tear and verified it with 4 commonly used microscopic assessment systems. The macroscopic grading system has 3 parameters, villous hypertrophy, hyperemia and density, which have been most frequently observed during arthroscopic surgery by a moderately experienced surgeon who has performed more than 1500 arthroscopic shoulder surgeries in last 5 years. Each of these parameters has also been frequently described in the literature about arthritic synovial inflammation [21, 37, 38]. We believe that these parameters are ubiquitous and easy to find and evaluate; thus, they would be adequate to represent the status of synovial inflammation found in patients with a full-thickness rotator cuff tear. We have developed a grading system with these 3 parameters that is feasible to use and correspond well with widely used microscopic assessment systems. This macroscopic grading system showed excellent inter- and intra-observer reliability, as shown with all of the ICCs above 0.8 [39]. Taken together, we suggest that the present macroscopic grading system would be a useful tool for the assessment of synovial inflammation in patients with a full-thickness rotator cuff tear and possibly in other shoulder diseases in which synovitis commonly occurs.

The macroscopic assessment showed that the severity of synovitis was significantly greater in the glenohumeral joint, 1.46 ± 0.59, than in the subacromial space, 0.89 ± 0.55 (*p* < 0.001). This is an unexpected and quite opposite finding to conventional thought because rotator cuff disease has long been considered a pathologic lesion in the subacromial space [14, 22, 23, 28]. Meanwhile, the cause of pain in rotator cuff tear has not been fully elucidated, and evidences in other diseases have shown that synovitis could be a determinant of pain and a predictor of cartilage destruction [21, 38, 40–43]. The present results along with the previous evidences suggest that pain in patients with rotator cuff tear might originate from the glenohumeral joint rather than, or at least as well as, from the subacromial space. In addition, they may provide a possible explanation regarding why glenohumeral joint degeneration often progresses in patients with rotator cuff tear. Gotoh et al. suggested this possibility with synovium harvested from the glenohumeral joint [17], and our results are consistent with theirs and further confirm them by comparing synovitis in the subacromial space. We expect that the current results would offer a new angle on synovitis in rotator cuff tear that could change the target, method and timing of treatment and call for further research.

The macroscopic assessment also demonstrated that the severity of synovitis varied considerably with respect to ROI in both the glenohumeral joint and the subacromial space. Generally, synovitis was most severe near the tear. In the glenohumeral joint, the macroscopic grade was higher in the posterior and anterior synovium than in the inferior synovium, which is away from the tear. In the subacromial space, the grades were higher in the lateral and anterior synovium than in the medial and posterior synovium, both of which are relatively distant from the most common tear location, the anterolateral aspect of the rotator cuff. In particular, the posterior synovium showed the lowest grades for all 3 parameters and for the macroscopic grade. These results are consistent with previous findings from knee joints with osteoarthritis [38, 44]. Lindblad et al. reported that inflammatory synovial changes were most intense near the cartilage and that signs of synovitis tapered with increasing distance from the cartilage, and they might be a specific feature of OA. One clinical implication from these results is that the regions near the tear should be managed first when performing interventions for reducing synovial inflammation, either through injection or surgical debridement, and that injection toward the inferior synovium in the glenohumeral joint and the posterior synovium in the subacromial space might be less effective.

The microscopic assessments, except for the Scanzello system, showed that synovitis in the glenohumeral joint was more severe than that in the subacromial space. Meanwhile, synovitis was not different between ROIs in the glenohumeral joint or in the subacromial space when measured with the 4 microscopic assessment systems. We think that these results are consistent with the results of the macroscopic assessment rather than in contrast to them. The parameters that differentiated the macroscopic grading between different ROIs in the glenohumeral joint were hyperemia and density, while hypertrophy was not different (Table 3). Except for the posterior synovium, which showed the lowest scores in all 3 parameters, the other 3 ROIs were not also different in the parameter of hypertrophy in the subacromial space. Considering that the microscopic assessments could not directly reflect hyperemia and density, these results are in line with the results of our macroscopic assessments as well as with previous results from studies of knee OA that reported that inflammatory synovial changes were microscopically indistinguishable irrespective of clinical diagnosis, duration, or activity [38, 45]. Therefore, the evidence suggests that the microscopic assessments alone would be inappropriate and lead to erroneous conclusions despite the high correlation among them, and that the macroscopic assessment should be performed with respect to adequately classified ROIs in the joint.

Limitations of the study are that it did not include or correlate clinical measures such as pain and shoulder function with the macroscopic measures of synovitis. However, the authors expect that the present study would be a useful basis for further studies and could facilitate them. Another limitation is that the macroscopic assessment was performed via arthroscopy, which is a minimally but nevertheless invasive procedure compared to non-invasive imaging techniques such as magnetic resonance imaging (MRI).

## Conclusions

We have suggested and validated a macroscopic assessment system for synovitis in rotator cuff disease. It showed excellent reliability and modest correlation with 4 commonly used microscopic assessment systems. The macroscopic assessment system well described characteristics of synovitis and could differentiate the severity of synovitis according to the location in both the glenohumeral joint and the subacromial space, which none of the microscopic assessment systems could do. Therefore, it could be a useful tool for investigating synovitis in rotator cuff disease, especially in clinics with no microscopic assessment system is available.

## Abbreviations

ROI: Regions-of-interest
H&E: Hematoxylin and eosin
ICC: Intraclass correlation coefficients
ANOVA: One-way analysis of ariance
