# Erratum to: Macroscopic and microscopic assessments of the glenohumeral and subacromial synovitis in rotator cuff disease

**DOI:** 10.1186/s12891-015-0833-6

**Published:** 2015-12-07

**Authors:** Chris H. Jo, Ji Sun Shin, Ji Eun Kim, Sohee Oh

**Affiliations:** Department of Orthopedic Surgery, SMG-SNU Boramae Medical Center, Seoul National University College of Medicine, Seoul, Korea; Department of Pathology, SMG-SNU Boramae Medical Center, Seoul National University College of Medicine, Seoul, Korea; Department of Biostatistics, SMG-SNU Boramae Medical Center, Seoul, Korea

## Erratum

After publication of the original article [1], the authors found that there was an error in describing "hyperemia", one of the three parameters of the macroscopic grading system of synovitis in the "Methods" section, an important description for the study.

In the final third of the second paragraph of the "Macroscopic assessment of synovitis with arthroscopy" subsection of the "Methods" section, the description of the "hyperemia grade" should not have included grade 2. Hyperemia has only 0 or 1 grade, and thus should have appeared as follows:

"Hyperemia represents the vascularity of synovial villi and was evaluated based on the redness of the villi; 0, pale and transparent to slightly reddish; 1, definitely red. If not apparent, hyperemia was determined with the harvested synovial tissue."
